# Hospitalisation with otitis media in early childhood and cognitive function in young adult life: a prevalence study among Danish conscripts

**DOI:** 10.1186/1471-2431-13-8

**Published:** 2013-01-15

**Authors:** Marie Mortensen, Rikke Beck Nielsen, Niels Fisker, Mette Nørgaard

**Affiliations:** 1 Department of Clinical Epidemiology, Aarhus University Hospital, Olof Palmes Allé 43-45, 8200, Aarhus N, Denmark; 2 H.C. Andersen Children’s Hospital, Odense University Hospital, Sdr. Boulevard 29, 5000, Odense C, Denmark

**Keywords:** Otitis media, Cognitive function, Educational level, Hearing impairment

## Abstract

**Background:**

Otitis media (OM) is a very common condition in children and occurs during years that are critical to the development of learning, literacy, and math skills. Therefore, among a large cohort of Danish conscripts, we aimed to examine the association between hospitalisation with OM in early childhood and cognitive function and educational level in early adulthood.

**Methods:**

We conducted a population-based prevalence study using linked data from healthcare databases and conscription records of Danish men born between 1977 and 1983. We identified all hospitalisations with OM before 8 years of age. Cognitive function was measured by the Boerge Prien validated group intelligence test (Danish Børge Prien Prøve, BPP). We adjusted for potential confounders with and without stratification by hearing impairment. Furthermore, we examined the association between hospitalisation with OM and the prevalence of having achieved a General Certificate of Secondary Education (GCSE), stratified by quartiles of BPP scores.

**Results:**

Of the 18 412 eligible conscripts aged 18–25 years, 1000 (5.5%) had been hospitalised with OM before age 8. Compared with conscripts without such a record, the adjusted prevalence ratio (PR) for a BPP score in the bottom quartile was 1.20 (95% confidence interval [CI]: 1.09–1.33). There was no major difference in the proportion of men with a GCSE and those without among those hospitalised with OM in early childhood. For men in the bottom and upper quartiles of BPP scores, the PRs for early childhood hospitalisation with OM were 0.89 (95% CI: 0.59–1.33) and 0.96 (95% CI, 0.88–1.05), respectively. Among men with severe hearing impairment, the proportion with a BPP score in the bottom quartile did not differ between those with and without an OM hospitalisation [PR = 1.01 (95% CI: 0.78**–**1.34)].

**Conclusions:**

Overall, we found that hospitalisation with OM in early childhood was associated with a slightly lower cognitive function in early adulthood. Hospitalisation for OM did not seem to influence the prevalence of GSCE when level of BPP was taken into account.

## Background

Acute otitis media (OM) is the most common infectious disease in young children, having occurred in 80% of all children by age 3 [1]. Furthermore, acute OM and OM with effusion (OME) are among the main reasons for early childhood consultations with general practitioners, and acute OM is one of the most common reasons for prescribing antibiotic treatment [2,3].

Since OM occurs in the critical years of language, literacy, and math skill development [4], OM in early childhood may affect cognitive function later in life. In a literature review of Monasta *et al.*, permanent hearing impairment was found to be a possible effect of OM in developing countries [5]. In a longitudinal cohort study of 74 children with early OM, Gravel *et al*. also found that mild conductive hearing impairment may influence auditory abilities in the longer term but only at high-frequency thresholds [6]. Moreover, in a prospective cohort study of 73 children with congenital deafness, Coletti *et al.* demonstrated improved auditory, speech language, and cognitive performances in children who received a cochlear implant prior to 12 months of age compared with children implanted later [7]. Children with mild to moderate permanent hearing loss are at risk for later effects on language and academic development [8]. In a meta-analysis Roberts *et al.* found no to very small effect of OM to speech and language development in most children [9]. These findings imply an association between hearing impairment and cognitive function.

However, the long-term consequences of OM are inadequately understood, and data on the association between OM in early childhood and cognitive function in adulthood are sparse and contradictory. For instance, Bennett *et al.* followed a birth cohort of 1000 children into their teens and found that a history of early middle ear disease appeared to affect reading ability, verbal intelligence quotient, and behavior problems [10]. In contrast, Zumach *et al.* found in a cohort study of 65 children that OM in early childhood was associated with hearing impairment, which decreased language comprehension and language skills at age 2, but not at age 7 [11]. Similarly, Grievink *et al*. found no correlation between the effects of OME and linguistic ability at age 7 in a cohort of 305 children [12]. In addition, the prospective study of 698 children by Johnson *et al.* found that prolonged OME was associated with decreased cognitive function at age 3, but that this association was not found by ages 5–7 [13]. Finally, in a prospective study of 241 children at 3 years of age, Paradise *et al.* found no correlation between the duration of OME and verbal aspects of cognition [14].

As cognitive function has been examined predominantly in early childhood and with school-age children, whether or not OM in early childhood and its possible impact on cognitive function also affects the level of education achieved is also not fully clarified [10-14]. Along these lines, a previous study by Welch and Dawes found no association between early childhood middle ear disease and adult measures of education [15].

Therefore, we examined the association between hospitalisation with OM in early childhood and cognitive function and educational level in young adult life using a large Danish army conscript data registry.

## Methods

We conducted a prevalence study based on registry data collected in the Fifth Military Conscription District of Denmark, which includes the former counties of North Jutland and Viborg (population approximately 700 000). We included all men born as singletons from January 1, 1977, to December 31, 1983, who registered in the Fifth Military Conscription District from 1997 to 2003. Evaluation at the military draft board is mandatory for all Danish men aged 18–20 years. Men must register with authorities in one of the country’s conscription districts, which were determined by their place of residence at age 18. During registration, men can report conditions that potentially preclude military service. Draft board physicians verify these reports and men with a verified condition are exempted from military duty without further examination. Documentation for the diagnosis leading to exemption is filed in the Conscript Registry and coded according to the 10th revision of the *International Classification of Diseases* (ICD-10) [16].

### Otitis media

We used the civil registration number (a unique personal identifier assigned at birth) to link data from the Conscript Registry to information on previous hospitalisations for OM recorded in the Danish National Registry of Patients (DNRP) [17]. The DNRP contains data on all hospitalisations in Denmark since 1977, including civil registration numbers and diagnoses coded according to the *International Classification of Diseases* eighth revision (ICD-8) until 1994, and the tenth revision thereafter [16].

We defined hospitalisation with OM as at least one hospital diagnosis of OM (ICD-8 codes 381 and 382) registered before the age of 8, since the prevalence of OM among Danish children is highest in this age group [18]. Thus, we included cases of OM with and without mastoiditis, acute OM, OME, and chronic suppurative OM. Hereafter, OM refers to any of these different types of OM.

### Cognitive function and educational level

All men who attend the evaluation at their military draft board must take a 45-minute group intelligence test, the Boerge Prien test (Danish Børge Prien Prøve, BPP), which has been used since 1957 by Danish military draft boards [19]. The test includes four time-limited subtests: letter matrices, verbal analogies, number series, and geometric figures. The single final score is the sum of correctly answered items (range: 0–78). BPP scores correlate to a large extent with scores in the Wechsler Adult Intelligence Scale (WAIS) (correlation coefficient = 0.82), although the BPP is a group test and the WAIS is an individually-administered test. Nevertheless, both tests are assumed to measure general intelligence [19]. From the conscript records, we also obtained information on whether the conscripts had achieved or were currently achieving the General Certificate of Secondary Education (GCSE), which is typically achieved at the age of 18 or 19 in Denmark.

### Covariates

We obtained information on gestational factors from the Danish Medical Birth Registry, which has tracked all births in Denmark since January 1, 1973 [20]. The information is derived from birth notification forms completed by midwives attending the birth. Variables include civil registration number, date and place of birth, gestational age, birth weight, maternal parity, and, since 1978, information on maternal marital status and Apgar score.

Given that permanent hearing impairment may be a consequence of OM in developing countries [5] and hearing impairment is also associated with cognitive function [7,8], we obtained information on hearing from conscript records. At the conscript examination an audiometric test is performed. The recording of the audiogram measurements are undertaken at frequencies 500, 1000, 2000, 3000, 4000, 6000 and 8000 Hz. Initial screening are carried out equivalent to 20 decibel [21]. Hearing is classified on a scale from 0–5 (severe hearing impairment to no hearing impairment). To improve the precision of the estimates, we collapsed the scale into severe hearing impairment (0–3), moderate hearing impairment (4), and no hearing impairment (5).

### Statistical analysis

We first constructed a box-and-whisker plot of BPP score distributions according to age at first hospitalisation for OM. We defined low cognitive function as a BPP score in the bottom quartile and used a log-binomial regression to estimate crude and adjusted prevalence ratios (PRs) for a BPP score in this quartile, according to age at first hospitalisation for OM [22]. PRs for a BPP score in the bottom quartile were estimated and stratified according to hearing impairment measured at the time of conscription examination.

The following possible confounding factors were included as identified from the literature: birth order (0, 1, 2, 3+), maternal age (≤20, 21–35, >35 years), marital status (married/unmarried), 5-minute Apgar score (<7, 7–10), gestational age, a composite variable derived from gestational age and birth weight [small for gestational age (SGA) or not] [23], and hearing score at conscription (severe, moderate, or no hearing impairment). In the regression analysis all variables were entered as sets of indicator variables. In a separate analysis we additionally adjusted for a previous diagnosis of febrile seizures or epilepsy. We examined PRs for OM hospitalisation and GCSE stratified into four quartiles by BPP score. To avoid loss of observations, missing values were estimated using multiple imputations, such that five imputed datasets were created and analysed with their averages serving as the estimates [24,25]. The regression model used for imputation included variables for gestational age, SGA or non-SGA, birth order, maternal age, marital status, and Apgar score 5-minutes after birth [26]. Confidence intervals (CI) around these estimates reflect uncertainty both about the value of the PRs and about the imputed values.

There was a lack of cognitive data on the exempted men because many of the conscripts (11.9%) were exempted from draft board examination. To quantify this potential selection bias, a sensitivity analysis was performed in which the regression analyses were repeated with imputation of BPP scores for all the 2470 exempted men. To assess the maximal impact of a potential selection bias, the regression analyses for the worst case scenario and best case scenario was repeated assuming that all exempt men had a BPP score in the bottom quartile and no exempt men had a BPP in the bottom quartile, respectively. Model-estimated PRs were compared with corresponding pooled Mantel-Haenszel (non-parametric) estimates. In an additional analysis, all men with a previous diagnosis of meningitis were excluded (68 men out of 18 412 in the study population).

Data were analysed using Stata software, version 11.2 (Stata, 4905 Texas, USA).

The study was approved by the Danish Data Protection Agency (http://www.datatilsynet.dk/english/, record no. 2011-41-5807). Data were obtained from the Conscript Registry, Danish Medical Birth Registry, and the DNRP. According to Danish legislation the study did not require permission from a Scientific Ethical Committee.

## Results

### Descriptive data

A total of 21 051 men born as singletons during the 1978–1983 period were registered in the Fifth Conscription District of Denmark. Of these, 161 (0.8%) had been hospitalised with OM after the age of 8. Of the eligible men, 2470 (11.7%) were exempt from draft board evaluation for health reasons. BPP scores were missing for 8 of the 18 420 non-exempt men (0.04%), leaving 18 412 men in the analysis (descriptive data are presented in Table 1). Of these, 1000 men (5.4%) had been hospitalised with OM before age 8.

**Table 1:** Characteristics of conscripts, who were evaluated at the draft board and had a Boerge Prien test (BPP)

	**Otitis media hospitalisation in childhood**		
**Variables**	**Yes**	**No**	**Total**
Men evaluated by the draft board, n (%)	1000 (5.4%)	17 412 (94.6%)	18 412 (100%)
BPP score, median (interquartile range)	42 (35–48)	44 (38–50)	44 (38–50)
Proportion with a BPP score in the bottom quartile (%)	312 (31.2%)	4370 (25.1%)	4682 (25.4%)
Birth weight in grams, median (interquartile range)	3450 (3125–3760)	3510 (3160–3875)	3500 (3150–3875)
**Gestational age,** no. (%)			
Preterm (<37 weeks)	53 (5.3%)	648 (3.7%)	701 (3.8%)
Term (31–41 weeks)	708 (70.8%)	12 762 (73.3%)	13 470 (73.2%)
Post-term (≥42 weeks)	66 (6.6%)	1209 (6.9%)	1275 (6.9%)
Missing	2793 (16.0%)	173 (17.3%)	2966 (16.1%)
**Small for gestational age,** no. (%)			
Yes	17 (1.7%)	141 (0.8%)	158 (0.9%)
No	808 (80.8%)	14 385 (82.6%)	15 193 (82.5%)
Missing	175 (17.5%)	2886 (16.6%)	3061 (16.6%)
**Apgar score at 5 minutes after birth,** no. (%)			
<7	6 (0.6%)	99 (0.6%)	105 (0.6%)
≥7	908 (90.8%)	16 077 (92.3%)	16 985 (92.2%)
Missing	86 (8.6%)	1236 (7.1%)	1322 (7.2%)
**Maternal age,** n (%)			
≤ 20 years	100 (10.0%)	1638 (9.4%)	1738 (9.4%)
21–35 years	852 (85.2%)	14 794 (84.9%)	15 646 (84.9%)
> 35 years	48 (4.8%)	980 (5.6%)	1028 (5.6%)
**Mothers’ marital status at subject′s birth,** n (%)			
Married	595 (59.5%)	11 033 (63.4%)	11 628 (63.2%)
Unmarried	341 (34.1%)	5366 (30.8%)	5707 (31%)
Missing	64 (6.4%)	1013 (5.8%)	1077 (5.9%)
**Birth order,** n (%)			
1	320 (32.0%)	6063 (34.8%)	6383 (34.7%)
2	368 (36.8%)	6148 (35.3%)	6516 (35.4%)
3	183 (18.3%)	3173 (18.2%)	3356 (18.2%)
4+	129 (12.9%)	2026 (11.6%)	2155 (11.7%)
Missing	0 (0%)	2 (0.01%)	2 (0.01%)
**Mode of delivery,** no. (%)			
Vaginal	796 (79.6%)	14 170 (81.4%)	14 966 (81.3%)
Caesarean	119 (11.9%)	1626 (9.3%)	1745 (9.5%)
Forceps or vacuum extraction	85 (8.5%)	1616 (9.3%)	1701 (9.2%)
**Hearing score at conscription,** no. (%)			
Severe hearing impairment	78 (7.8%)	754 (4.3%)	832 (4.5%)
Moderate hearing impairment	274 (27.4)	3902 (22.4%)	4176 (22.7%)
No hearing impairment	641 (64.1%)	12 653 (72.7%)	13 294 (72.2%)
Missing	7 (0.7%)	103 (0.6%)	110 (0.6%)

The prevalence of exemption prior to draft board evaluation was 20% for men hospitalised with OM in early childhood and 11% for men with no record of a hospitalisation with OM.

**Figure 1:**
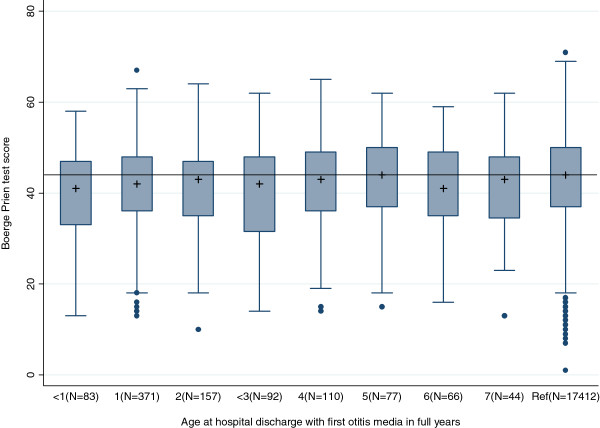
**BPP scores by age at first hospitalisation diagnosis with otitis media (OM) in Danish conscripts.** The group comprised conscripts without a hospitalisation with OM. The cross marks the median of a BPP score, and the box marks the upper and lower quartiles. The whiskers extend 1.5 times the interquartile range. Observations outside that range are plotted individually. The horizontal line shows sample median BPP, which equals 44.0.

For the non-exempt conscripts, the median BPP scores were 42 (interquartile range: 35–48) for men with an OM hospitalisation and 44 (interquartile range: 38–50) for men without an OM hospitalisation (Table 1). Most cases of hospitalisation with OM (37%) occurred in the second year of life. The median BPP scores varied between 41 and 44 (see Figure 1) when stratified according to age at first hospitalisation with OM.

### Prevalence ratios for Boerge Prien test (BPP) scores

The prevalences of BPP scores in the bottom quartile (score ≤37) were 31.2% (312/1000) for men hospitalised with OM in early childhood and 25.1% (4370/17 412) for men without such a hospitalisation.

Among men without hearing impairment at the time of draft board evaluation, those who had been hospitalised with OM in early childhood had a 15% greater risk of a BPP score in the bottom quartile than the other men in the study (Table 2). Among men with severe hearing impairment (7.9% of men with OM hospitalisation and 4.4% of the men without), the proportions with a BPP score in the bottom quartile were similar [PR = 1.01 (95% CI: 0.78–1.34)].

**Table 2:** Distribution of Boerge Prien test (BPP) scores and prevalence ratios, stratified according to hearing score

	**Otitis media hospitalisation in childhood**	
	**Yes**	**No**
**Severe hearing impairment (hearing score 0–3)**		
Number, %	78 (7.9%)	754 (4.4%)
Median BPP (quartile)	40 (32–47)	39 (33–46)
Men with a BPP in the bottom quartile	33 (42.3%)	313 (41.5%)
PR for BPP in the bottom quartile, 95% CI	1.01 (0.78–1.34)	1 (reference)
**Moderate hearing impairment (hearing score 4)**		
Number, %	274 (27.6%)	3902 (22.5%)
Median BPP (quartile)	41 (33–46)	43 (38–49)
Men with a BPP in the bottom quartile	105 (38.3%)	1107 (28.4%)
PR for BPP in the bottom quartile, 95% CI	1.35 (1.15–1.58)	1 (reference)
**No hearing impairment (hearing score 5)**		
Number, %	641 (64.6%)	12 653 (73.1%)
Median BPP (quartile)	43 (38–49)	44 (38–50)
Men with a BPP in the bottom quartile	169 (26.4%)	2908 (22.9%)
PR for BPP in the bottom quartile, 95% CI	1.15 (1.01–1.32)	1 (reference)
**Total**	993 (100%)	17 309 (100%)

Compared with men without childhood hospitalisation for OM, the adjusted PR for a BPP score in the bottom quartile for men with an OM hospitalisation was 1.20 (95% CI: 1.09–1.33) (Table 3). None of the potential confounding variables noticeably affected the PR estimates, either separately or in combination. Including men who did not come to the conscription examination by imputing their BPP values did not changes the estimates.

**Table 3:** Crude and adjusted prevalence ratios for Boerge Prien test (BPP) scores overall and according to age

	**Total men with BPP score in the bottom quartile**		**PR for BPP score in the bottom quartile**	
		**n (%)**	**n (%)**	
			**Crude PR**	Adjusted* PR
**Hospitalisation with OM**				
No	17 412	4370 (25.1)	1	1
Yes	1000	312 (31.2)	1.24 (1.13–1.37)	1.20 (1.09–1.33)
**Age at first hospitalisation with OM**				
< 1 year	83	30 (36.1)	1.44 (1.08–1.92)	1.38 (1.04–1.83)
1–2 years	528	163 (31.0)	1.23 (1.07–1.40)	1.17 (1.03–1.33)
3–5 years	279	85 (30.5)	1.22 (1.02–1.45)	1.24 (1.03–1.49)
6–7 years	110	34 (30.9)	1.23 (0.93–1.63)	1.17 (0.89–1.54)

*Based on log binomial regression using multiple imputation for the following variables: gestational age (<37, 37–41, >41), gestational age adjusted birth weight (SGA, non-SGA), birth order (1,2,3,4+), maternal age (20, 21–35, >35 years), marital status (married/unmarried), and Apgar score 5-minutes after birth (<7, ≥ 7).

There was no major variation in the prevalence of GCSE by quartiles of BPP scores among men with and without an OM hospitalisation in early childhood, as the PR was 0.89 (95% CI: 0.59–1.33) for those in the bottom quartile of BPP scores and 0.96 (95% CI: 0.88–1.05) for those in the upper quartile (Table 4). Excluding men who previously had been diagnosed with meningitis also did not change the estimates.

**Table 4:** Prevalence of GCSE among conscripts with and without hospitalisation for otitis media (OM), stratified by Boerge Prien test (BPP) scores

	**With GCSE**		**Without GCSE**		
	**With OM n,%**	**Without OM n,%**	**With OM n,%**	**Without OM n,%**	**PR**
**BPP ≤37**	23 (7.4)	363 (8.3)	288 (92.6)	4000 (91.7)	0.89 (0.59–1.33)
**BPP 38–44**	89 (28.7)	1481 (31.2)	221 (71.3)	3272 (68.8)	0.92 (0.77–1.10)
**BPP 45–50**	104 (52.8)	2389 (55)	93 (47.2)	1954 (45)	0.96 (0.84–1.10)
**BPP >50**	134 (74.0)	3022 (76.9)	47 (26)	906 (23.1)	0.96 (0.88–1.05)
**Total**	7605 (41.4)		10 781 (58.6)		

Assuming the worst case scenario, with all exempt men having a BPP score in the bottom quartile, resulted in a PR of 1.29 (95% CI, 1.21 to 1.38), while the surplus for the best case scenario was a PR of 1.09 (95% CI, 0.99 to 1.21).

## Discussion

In this study of 18 412 conscripts, we examined long-term cognitive outcomes after hospitalisation for OM before age 8. Overall, we found that hospitalisation with OM in early childhood was associated with a slightly lower cognitive function in early adulthood except in men with severe hearing impairment at the time of draft board evaluation. We found no association between hospitalisation for OM in early childhood and GCSE beyond that explained by level of cognitive function.

Our study thus extends the findings by Bennett *et al.* who found that some developmental sequelae of OME could continue into the early teens [10]. Our findings are in contrast to the studies of Roberts *et al.,* Zumach *et al.,* Grievink *et al.,* and Johnson *et al.*[9,11-13] who could not detect any negative consequences of OM and OME by 7 years of age. However, these studies were all smaller than our population-based study. Additionally, our findings also agree with those of Welch and Dawes, which indicate that OM in early childhood does not affect the level of education attained [15].

We only focused on those admitted to the hospital for OM in early childhood. By age 3 it is estimated that 80% of all children have experienced at least one episode of OM [1], though only a minority of these children are admitted to the hospital. Admitted cases presumably represent more severe cases of OM. Still, other factors may influence whether or not a child is admitted to the hospital for OM, such as socioeconomic status (SES). Unfortunately, we had limited information on SES and were thus unable to determine whether our observed association could be explained by severity of OM or by other factors influencing hospitalisation for OM. In addition, hearing impairment is probably also related to the severity of OM [5]. Yet, when we stratified according to hearing impairment at conscription examination, we found no differences in BPP among men hospitalised for OM in early childhood. This could indicate that the correlation between OM and low BPP cannot be explained by hearing loss alone.

Our study has other strengths and limitations. The possibility of certain types of selection biases was reduced by independent collection of data on OM and BPP scores in a population with tax-funded free access to health care and free hospitalisations, if indicated. Still, we examined outcomes among men who survived and stayed in the region to conscription age, rather than following a birth cohort. We also lacked data on the cognitive function of men exempt from conscript examination; if some were exempt because of conditions related to OM and lower cognitive function, we may have underestimated the association between hospitalisation for OM and cognitive function in young adulthood [19]. However, imputing BPP scores for exempt men did not change our results. The sensitivity analysis showed that the risk of significant selection bias, because of the exempt men was minimal. In addition, our study relied on hospitalisation diagnoses, which may not have been accurately registered [27]. Misclassification of the OM diagnosis could have biased our relative estimates toward the null. Moreover, we were able to control for several gestational factors, but we lacked information on variables related to maternal lifestyle, such as smoking, alcohol intake, and medication use. We also lacked information on the home environment, day-care attendance, and further social factors of the conscripts as children, all of which may be potential confounding factors [10,28-31]. Although adjustment for measured confounders led to only minor changes in our estimates, unmeasured confounding can not be excluded as a potential explanation for the associations found in this study.

## Conclusion

Overall, we found a slightly lower cognitive function in early adulthood among men who were hospitalised with OM in early childhood. Further, we found no association between hospitalisation with OM and GCSE when the level of BPP was taken into account.

## Endnote

^a^General Certificate of Secondary Education: is referring to the Danish Upper Secondary School Leaving Examination.

## Abbreviations

BPP: Boerge Prien test (Danish Børge Prien Prøve); DNRP: Danish National Registry of Patients; GCSE: General Certificate of Secondary Education^a^; ICD: International Classification of Diseases; PR: Prevalence ratio; OM: Otitis media; OME: Otitis media with effusion; SES: Socioeconomic status; WAIS: Wechsler Adult Intelligence Scale.

## Competing interests

The authors declare that they have no competing interests and no financial relations to disclose that are relevant to this article.

## Authors’ contributions

MM, RBN, NF, and MN contributed to the conception and design of the study. MM, RBN, and MN contributed to the analysis of the data. All authors contributed to the interpretation of the data, drafting or revising of the manuscript, and final approval for publication. MM and MN are the guarantors.

## Pre-publication history

The pre-publication history for this paper can be accessed here:

http://www.biomedcentral.com/1471-2431/13/8/prepub
